# Identification of routine blood derived hematological and lipid indices in ILD through machine learning; a retrospective case-control study

**DOI:** 10.3389/fmed.2025.1633713

**Published:** 2025-10-09

**Authors:** Lichen Zhu, Yu Fu, Linchao Zhu, Yimin Yao, Li Chen

**Affiliations:** ^1^Artificial Intelligence and Big Data Center, The First Affiliated Hospital of Zhejiang Chinese Medical University (Zhejiang Provincial Hospital of Chinese Medicine), Hangzhou, China; ^2^The First School of Clinical Medicine, Zhejiang Chinese Medical University, Hangzhou, China; ^3^Department of Laboratory Medicine, The First Affiliated Hospital of Zhejiang Chinese Medical University (Zhejiang Provincial Hospital of Chinese Medicine), Hangzhou, China

**Keywords:** interstitial lung disease, routine blood test, inflammatory-metabolic indices, machine learning, random forest, clinical research

## Abstract

**Introduction:**

Interstitial lung disease (ILD) comprises various disorders marked by pulmonary inflammation and fibrosis. Early diagnosis and risk prediction are vital for improving patient outcomes.

**Methods:**

We retrospectively analyzed 603 patients who had visited the Hubin Campus between January 2022 and April 2025, employing a 1:2 case-control design with age- and gender-matched groups. We collected clinical information, complete blood count data, lipid metabolism indicators, and various derived indices.

**Conclusion:**

Six key markers were identified through three machine learning algorithms (LassoCV, SVMREFCV, and Boruta): neutrophil percentage, lymphocyte percentage, monocyte percentage, hemoglobin, and two novel ratios - neutrophil-to-HDL-C and lymphocyte-to-HDL-C. The random forest model outperformed seven other machine learning approaches, with AUC values of 0.868 (validation set), 0.885 (test set), and 0.849 (external cohort), demonstrating consistent predictive accuracy.

**Discussion:**

Based on these findings, we developed an online prediction tool to assist primary care clinicians in assessing the risk of ILD in suspected cases. Our results indicate that the random forest model exhibits high accuracy and clinical utility for early ILD prediction, providing a novel tool and methodology for early diagnosis and intervention. Future studies will focus on further optimizing the model and validating it in larger multicenter cohorts.

## 1 Introduction

Interstitial lung disease (ILD) encompasses various disorders involving lung tissue inflammation and scarring, with complex etiologies, diverse clinical manifestations, and significant prognostic variability (1). Due to aging populations and increasing environmental pollution, ILD incidence has risen significantly, emerging as a major global health challenge. Although considerable progress has been made in ILD diagnosis and treatment in recent years, the incomplete understanding of its pathological mechanisms continues to pose substantial challenges for early diagnosis and personalized therapy. Therefore, in-depth investigation of ILD pathogenesis and identification of effective biomarkers are of paramount importance for improving patient outcomes.

Currently, ILD diagnosis primarily relies on high-resolution computed tomography (HRCT) and pulmonary function tests, but these methods have limitations including radiation exposure, high costs, and operational complexity. Blood biomarkers (including KL-6, -D and MMPs) (2–4) are increasingly used because they’re non-invasive, convenient, and reproducible. Research shows that both systemic inflammation and immune dysregulation contribute significantly to ILD development, driving interest in related blood biomarkers (5, 6). However, most existing studies concentrate on single or limited combinations of biomarkers, lacking comprehensive analysis and integration of multidimensional blood parameters, which hinders a complete reflection of ILD’s complex pathophysiology.

Although prior investigations have linked single hematological indicators to ILD (7, 8), a systematic appraisal of composite, blood-derived inflammatory indices—such as the systemic inflammation response index (SIRI) and the systemic immune-inflammation index (SII)—is still lacking. Second, conventional statistical methods employed in most studies are inadequate for handling high-dimensional data and non-linear relationships, constraining biomarker screening and diagnostic association model development. Finally, the absence of large-scale, multicenter validation studies compromises the reliability and generalizability of findings. Therefore, there is an urgent need to employ advanced machine learning algorithms combined with multidimensional blood parameters to develop efficient and accurate ILD prediction models.

This retrospective cohort study enrolled 603 patients (including 201 ILD cases) from three campuses of Zhejiang Provincial Hospital of Traditional Chinese Medicine. We collected multidimensional data encompassing complete blood counts, lipid metabolism indicators, and various blood-derived parameters, using least absolute shrinkage and selection operator (LASSO) regression to identify ILD-associated features. Innovatively, this study applied eight machine learning algorithms (including XGBoost, logistic regression, and LightGBM) to construct ILD prediction models. Model performance was evaluated through calibration plots, sensitivity, specificity, accuracy, predictive values, and area under the curve (AUC), with an online prediction tool developed to provide primary care physicians with convenient ILD risk assessment.

This study aims to integrate multidimensional blood parameters using advanced machine learning algorithms to develop accurate ILD prediction models and corresponding online tools. The findings may offer novel approaches for early diagnosis and personalized treatment of ILD, while serving as a reference for biomarker research in other complex diseases. By bridging key research gaps through machine learning analysis of multidimensional biomarkers, this study aims to transform ILD diagnosis and treatment paradigms, potentially enhancing both survival rates and patients’ daily functioning.

## 2 Materials and methods

We conducted a retrospective case–control study among individuals who underwent chest CT at Hubin Campus between January 2022 and April 2025. A total of 603 subjects were enrolled: 201 with interstitial lung disease (ILD) and 402 without. The diagnosis of all ILD cases was established by a multidisciplinary team (MDT) consensus that included at least three experts from pulmonology, radiology, rheumatology, and pathology, based on integrated evaluation of HRCT, pulmonary-function testing, and targeted serum autoantibody panels. The ILD cohort included 22 IPF, 175 CTD-ILD, and 4 other subtypes. The non-ILD cohort was selected from contemporaneous CT recipients. This group comprised available connective-tissue-disease (CTD) patients without ILD and a representative subset of other pulmonary conditions, all explicitly adjudicated as free of interstitial lung disease. They were frequency-matched 1:2 to the ILD group on age (±3 years) and sex. Among these controls, 106 had underlying connective tissue disease but no evidence of ILD, and 296 had other pulmonary disorders (COPD, *n* = 219; asthma, *n* = 40; pulmonary edema, *n* = 37). Absence of ILD was independently verified by CT and by a respiratory physician, and blood samples were collected during the same outpatient visit or hospitalization.

Additionally, we collected data from 288 patients at two other campuses for external validation of the model. We extracted patient data (electronic medical records and laboratory results) from the hospital information system. Inclusion criteria were: (1) complete clinical data availability, (2) complete blood count and lipid panel data, and (3) age exceeding 50 years. The exclusion criteria included: (1) active infection, (2) concurrent diagnosis of malignant tumors or severe hematological disorders, and (3) on lipid-lowering medication. The relevant exclusion criteria and flow chart are shown in Supplementary Figure 1.

We systematically collected baseline clinical characteristics from enrolled patients, including demographic information (age, sex), comorbidities, HRCT findings, and results from standard peripheral blood tests. Our analysis included complete blood count parameters (leukocyte counts with differentials, erythrocyte indices, and platelet measurements) along with lipid metabolism markers (triglycerides [TG], total cholesterol [CHOL], high-density lipoprotein cholesterol [HDL-C], and low-density lipoprotein cholesterol [LDL-C]). All blood-derived data were collected within 7 days after the diagnosis of ILD. This timing ensured that the results reflected early inflammation and metabolic status. Full parameter definitions are provided in Supplementary Table 1.

We calculated the following derived hematological indices: neutrophil-to-lymphocyte ratio (NLR), derived neutrophil-to-lymphocyte ratio (dNLR), monocyte-to-lymphocyte ratio (MLR), neutrophil-monocyte-to-lymphocyte ratio (NMLR), systemic inflammation response index (SIRI), systemic immune-inflammation index (SII), neutrophil-to-HDL cholesterol ratio (NHR), lymphocyte-to-HDL cholesterol ratio (LHR), monocyte-to-HDL cholesterol ratio (MHR), platelet-to-HDL cholesterol ratio (PHR), and non-HDL-to-HDL cholesterol ratio (NHHR). The calculation is as follows: NLR = neutrophil counts (10^9^/L)/lymphocyte count (10^9^/L), dNLR = Neutrophil count (10^9^/L)/(white blood cell count-lymphocyte count) (10^9^/L), MLR = monocyte count (10^9^/L)/lymphocyte count (10^9^/L), NMLR = (monocyte count + neutrophil count) (10^9^/L)/lymphocyte count (10^9^/L), SIRI = neutrophil count(10^9^/L) monocyte count (10^9^/L)/lymphocyte count (10^9^/L), SII = platelet count (10^9^/L) neutrophil count (10^9^/L)/lymphocytecount (10^9^/L). NHR = Neutrophil count (10^9^/L)/HDL cholesterol (mmol/L); LHR = lymphocyte count (10^9^/L)/HDL cholesterol (mmol/L); MHR = monocyte count (10^9^/L)/HDL cholesterol (mmol/L); PHR = PLT (10^9^/L)/HDL cholesterol (mmol/L); NHHR = [cholesterol (mmol/L) – HDL

cholesterol (mmol/L)]/HDL cholesterol (mmol/L). Based on HRCT findings, the 603 patients were divided into two groups: interstitial lung disease and normal controls.

Statistical analysis was performed using the Beckman Colter DxAI platform (https://www.xsmartanalysis.com/beckman/login). Least absolute shrinkage and selection operator (LASSO) regression was employed to identify factors associated with interstitial lung disease. We evaluated eight machine learning models (listed in Methods) using calibration plots, assessing sensitivity, specificity, accuracy, predictive values, and AUC. After randomly allocating 15.0% of samples as a hold-out test set, we performed 2-fold cross-validation on the remaining 85% (50% training, 50% validation per fold). The validation set achieved an AUC of 0.915 ± 0.034. The final model demonstrated an AUC of 0.885 ± 0.028 and accuracy of 0.868 in the test set, allowing identification of the optimal machine learning model. The selected model was subsequently validated in an independent external test cohort.

SPSS Modeler (version 16.0) and R (version 4.2.3) were employed in this study. For continuous data, we used *t*-tests (normal distributions) or Wilcoxon tests (non-normal distributions); for categorical data, we employed chi-square tests. The *t*-test was applied to data with normal distribution and homogeneity of variance, whereas the Wilcoxon signed-rank test was used for non-normally distributed or heteroscedastic variance. LASSO regression analysis was performed to identify predictors for ILD and to evaluate their predictive performance via receiver operating characteristic (ROC) curves. The statistical significance threshold was set at *P* < 0.05.

## 3 Results

### 3.1 Baseline features

Table 1 presents the baseline characteristics of 603 patients. Among these patients, 201 (33.3%) were diagnosed with interstitial lung disease, including 92 females (45.8%) and 109 males (54.2%). The control group included 402 patients (66.7%), comprising 174 females (43.3%) and 228 males (56.7%). In this study, no significant intergroup differences were observed in age (*P* = 0.623), sex (*P* = 0.622), monocyte percentage (MO%, *P* = 0.214), basophil count (BA, *P* = 0.111), platelet count (PLT, *P* = 0.832), or platelet distribution width (PDW, *P* = 0.053). All remaining measured parameters demonstrated statistically significant intergroup differences (*P* < 0.05).

**TABLE 1:** Baseline characteristics of the two groups of patients.

Characteristics		Normal control (*n* = 402)	ILD group (*n* = 201)	*P*-value
Sex, *N* (%)	Female	174 (43.3%)	92 (45.8%)	0.622
	Male	228 (56.7%)	109 (54.2%)	
Age, median [IQR]		70.000 [64.500;77.000]	70.000 [64.000;77.000]	0.623
**Variable category**				
WBC, median [IQR]		5.850 [4.900;6.900]	7.000 [5.800;8.500]	<0.001
NE%, median [IQR]		56.550 [50.700;61.600]	66.500 [57.500;76.000]	<0.001
LY%, median [IQR]		32.900 [28.200;38.100]	26.800 [19.700;32.700]	<0.001
MO%, median [IQR]		7.200 [6.200;8.200]	8.000 [6.500;9.400]	<0.001
EO%, median [IQR]		2.100 [1.325;3.000]	2.200 [1.300;4.300]	0.214
BA%, median [IQR]		0.600 [0.400;0.800]	0.500 [0.300;0.700]	<0.001
NE, median [IQR]		3.300 [2.600;4.000]	4.400 [3.300;6.700]	<0.001
LY, median [IQR]		1.900 [1.500;2.375]	1.700 [1.300;2.200]	0.001
MO, median [IQR]		0.400 [0.300;0.500]	0.600 [0.400;0.700]	<0.001
EO, median [IQR]		0.120 [0.070;0.190]	0.140 [0.080;0.250]	0.006
BA, median [IQR]		0.040 [0.020;0.050]	0.030 [0.020;0.050]	0.111
RBC, median [IQR]		4.545 [4.272;4.820]	4.150 [3.720;4.490]	<0.001
HGB, median [IQR]		141.000 [133.000;149.000]	127.000 [112.000;138.000]	<0.001
RDW, median [IQR]		13.300 [13.000;13.700]	13.800 [13.200;14.600]	<0.001
PLT, median [IQR]		209.000 [172.000;243.000]	209.000 [170.000;255.000]	0.832
PDW, median [IQR]		16.800 [16.500;17.200]	16.700 [16.300;17.200]	0.053
TG, median [IQR]		1.295 [0.950;1.750]	1.130 [0.890;1.560]	0.009
CHOL, median [IQR]		4.775 [4.152;5.395]	4.380 [3.570;5.110]	<0.001
HDLC, mean (±SD)		1.565 (0.300)	1.299 (0.359)	<0.001
LDLC, median [IQR]		2.615 [2.015;3.128]	2.425 [1.703;2.960]	0.006
NLR, median [IQR]		1.714 [1.337;2.197]	2.571 [1.889;3.700]	<0.001
dNLR, median [IQR]		0.848 [0.818;0.872]	0.875 [0.822;0.930]	<0.001
MLR, median [IQR]		0.222 [0.176;0.284]	0.314 [0.241;0.421]	<0.001
NMLR, median [IQR]		1.951 [1.553;2.462]	2.818 [2.188;4.250]	<0.001
SIRI, median [IQR]		0.722 [0.506;1.023]	1.511 [0.911;2.444]	<0.001
SII, median [IQR]		353.920 [256.170;481.996]	555.929 [316.800;817.765]	<0.001
MHR, median [IQR]		0.271 [0.207;0.347]	0.476 [0.325;0.640]	<0.001
NHHR, median [IQR]		1.206 [1.135;1.334]	1.282 [1.184;1.423]	<0.001
LHR, median [IQR]		1.233 [0.933;1.597]	1.364 [1.000;1.800]	0.007
PHR, median [IQR]		131.816 [109.233;165.622]	158.974 [121.477;212.605]	<0.001
NHR, median [IQR]		2.116 [1.592;2.763]	3.750 [2.386;5.294]	<0.001

### 3.2 Identification of feature factors associated with interstitial lung disease

**FIGURE 1:**
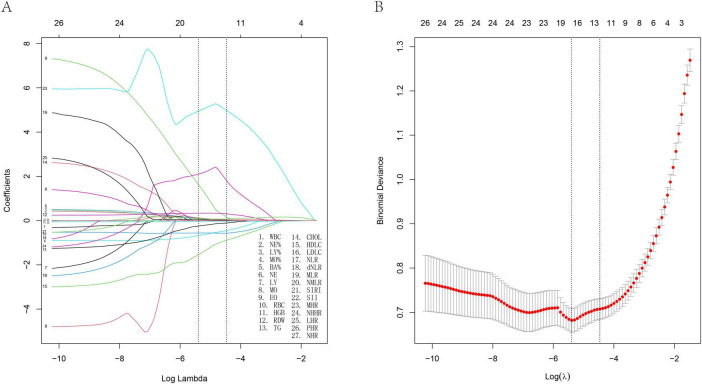
Least absolute shrinkage and selection operator (LASSO) regression analysis and 10-fold cross-validation of risk factors associated with ILD. **(A)** Sixteen non-zero coefficient risk factors were identified using the LASSO method. **(B)** Coefficient plot of generated log (λ) sequence.

**FIGURE 2:**
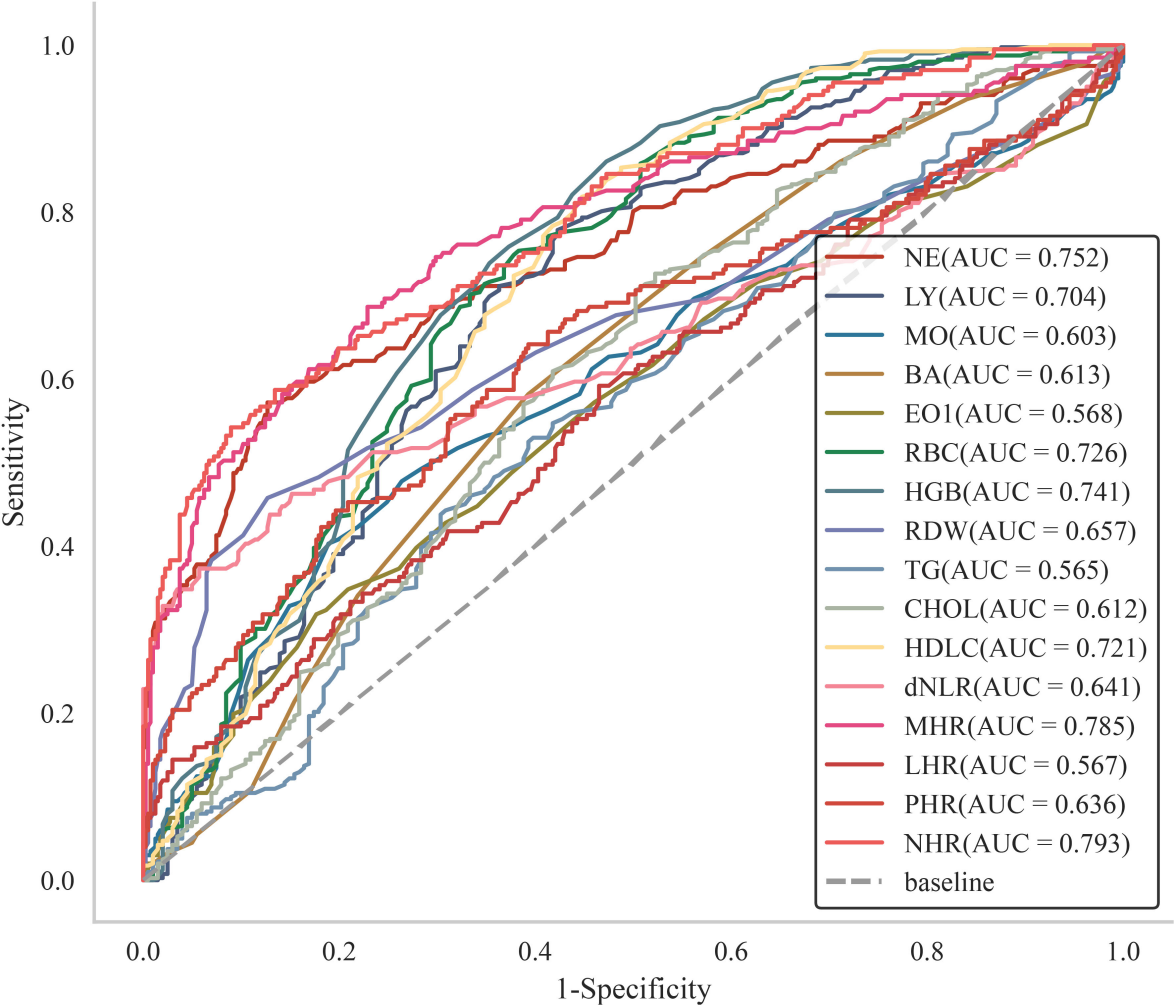
Subject operating characteristic (ROC) curves with different factors predicting the occurrence of ILD.

Using LASSO regression analysis, this study identified multiple factors associated with interstitial lung disease risk, as detailed in Table 1. The analysis identified sixteen significant biomarkers associated with interstitial lung disease (Figure 1), including neutrophil percentage (NE%), lymphocyte percentage (LY%), and 14 other hematological and biochemical parameters. Subsequently, we assessed the diagnostic performance of these factors by calculating their area under the curve (AUC) values through ROC analysis (Figure 2). The AUC values ranged from 0.565 to 0.793, with neutrophil-to-high density lipoprotein cholesterol ratio (NHR) showing the highest predictive value (AUC = 0.793) and eosinophil count (EO) the lowest (AUC = 0.568). Complete results are presented in Figure 2.

### 3.3 ML algorithm for feature identification

**FIGURE 3:**
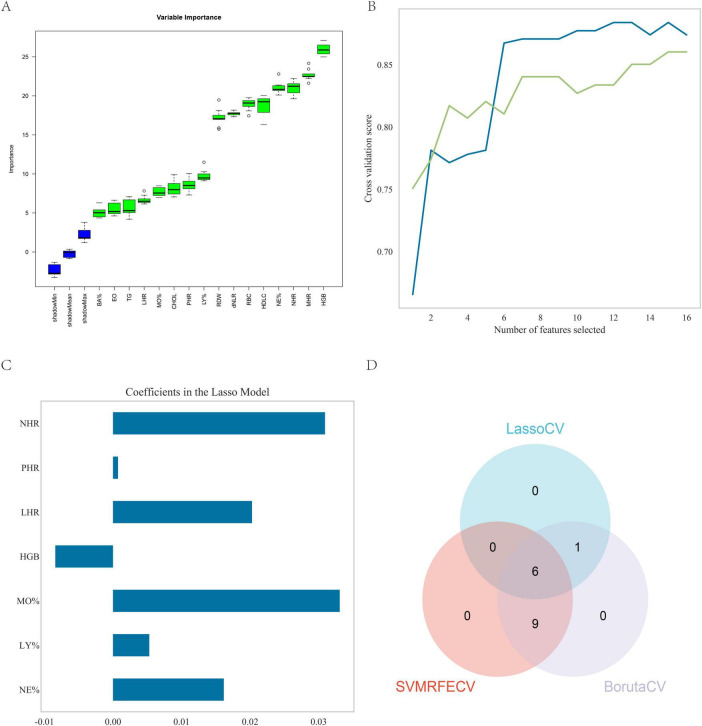
Feature screening. **(A)** Sixteen factors were selected using the Boruta method; **(B)** fifteen factors were selected using the SVMREFCV method; **(C)** seven factors were selected using the LassoCV method; **(D)** venn diagram of the three machine learning algorithms.

The algorithms LassoCV, SVMREFCV, and Boruta were employed to identify biomarkers, with results shown in Figures 3A–C respectively, and the resulting Venn diagram (Figure 3D) was generated using R. The intersection of results from all three algorithms revealed six overlapping biomarkers were identified: neutrophil percentage (NE%), lymphocyte percentage (LY%), monocyte percentage (MO%), hemoglobin (HGB), LHR, and NHR.

### 3.4 Optimal model identification

**FIGURE 4:**
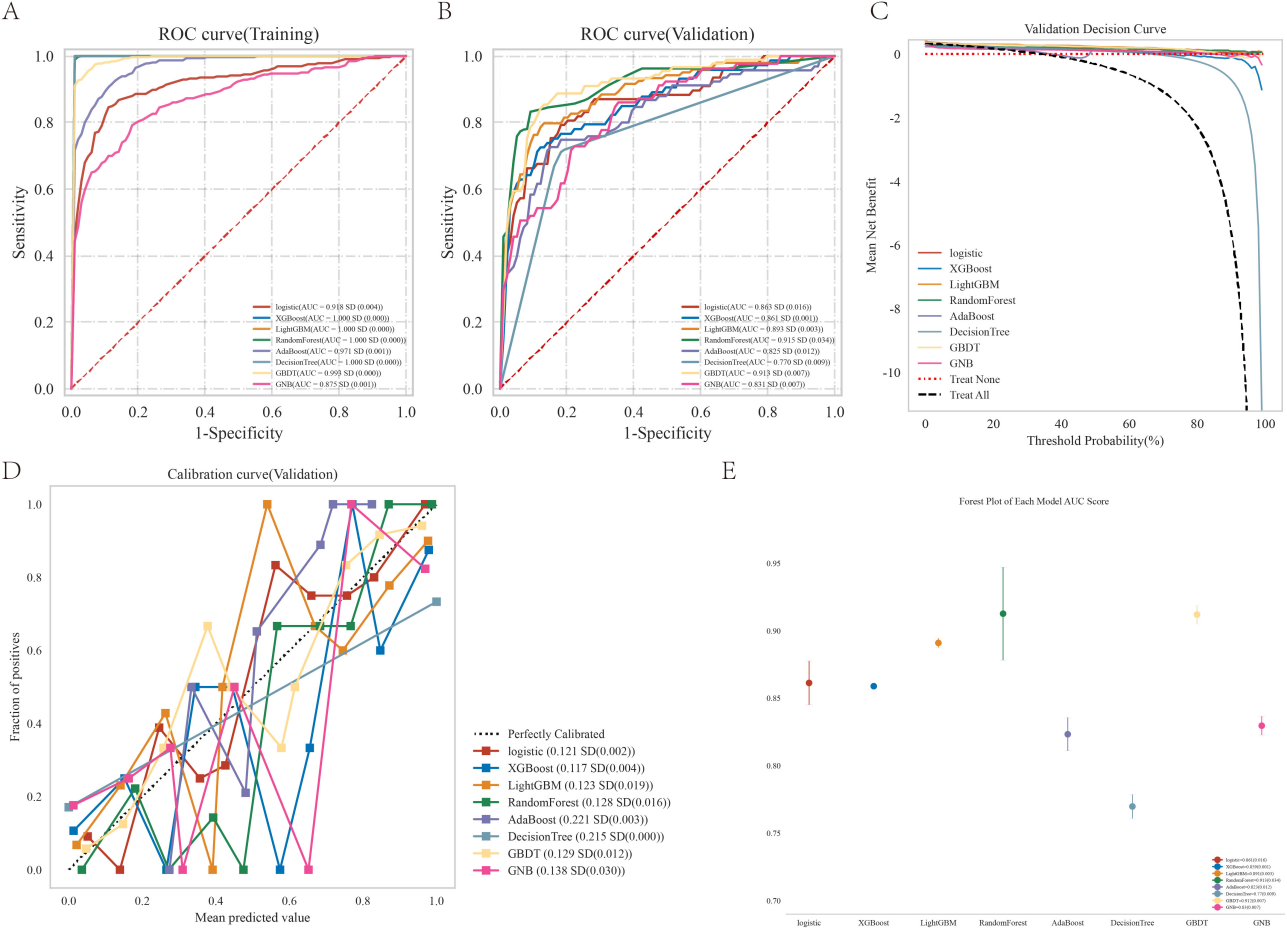
Performance comparison between multiple models. **(A)** ROC curve for the test cohort; **(B)** ROC curve for the validation cohort; **(C)** decision curve of the machine learning model; **(D)** calibration curve for the specific machine learning model; **(E)** forest area (AUC) in each area under the curve.

The random forest model demonstrated superior predictive accuracy among eight evaluated models (Figure 4 and Table 2), with AUC values of 0.868 and 0.885 in validation and test phases respectively. This performance was further supported by calibration curve analysis and decision curve analysis, confirming both model robustness and clinical applicability.

**TABLE 2:** The diagnostic effect of the eight classification models in the training and validation cohorts.

Classifier	Cohorts	AUC	Cutoff	Accuracy	Sensitivity	Specificity	Positive predictive value	Negative predictive value	F1
Logistic	Training	0.918	0.342	0.877	0.846	0.892	0.799	0.919	0.822
	Validation	0.863	0.342	0.822	0.727	0.867	0.719	0.872	0.723
XGBoost	Training	1.000	0.840	1.000	1.000	1.000	1.000	1.000	1.000
	Validation	0.861	0.840	0.843	0.660	0.924	0.794	0.863	0.716
LightGBM	Training	1.000	0.811	1.000	1.000	1.000	1.000	1.000	1.000
	Validation	0.893	0.811	0.777	0.515	0.974	0.931	0.734	0.657
RandomForest	Training	1.000	0.500	0.992	0.997	0.989	0.979	0.998	0.988
	Validation	0.915	0.500	0.880	0.781	0.928	0.831	0.901	0.803
AdaBoost	Training	0.971	0.494	0.894	0.923	0.881	0.786	0.960	0.849
	Validation	0.825	0.494	0.798	0.738	0.835	0.729	0.840	0.732
DecisionTree	Training	1.000	1.000	1.000	1.000	1.000	1.000	1.000	1.000
	Validation	0.770	1.000	0.789	0.712	0.828	0.667	0.853	0.687
GBDT	Training	0.993	0.328	0.960	0.955	0.962	0.926	0.978	0.939
	Validation	0.913	0.328	0.847	0.854	0.843	0.763	0.909	0.805
GNB	Training	0.875	0.025	0.813	0.795	0.822	0.693	0.889	0.740
	Validation	0.831	0.025	0.736	0.684	0.763	0.585	0.833	0.627

### 3.5 Random forest model analysis

**FIGURE 5:**
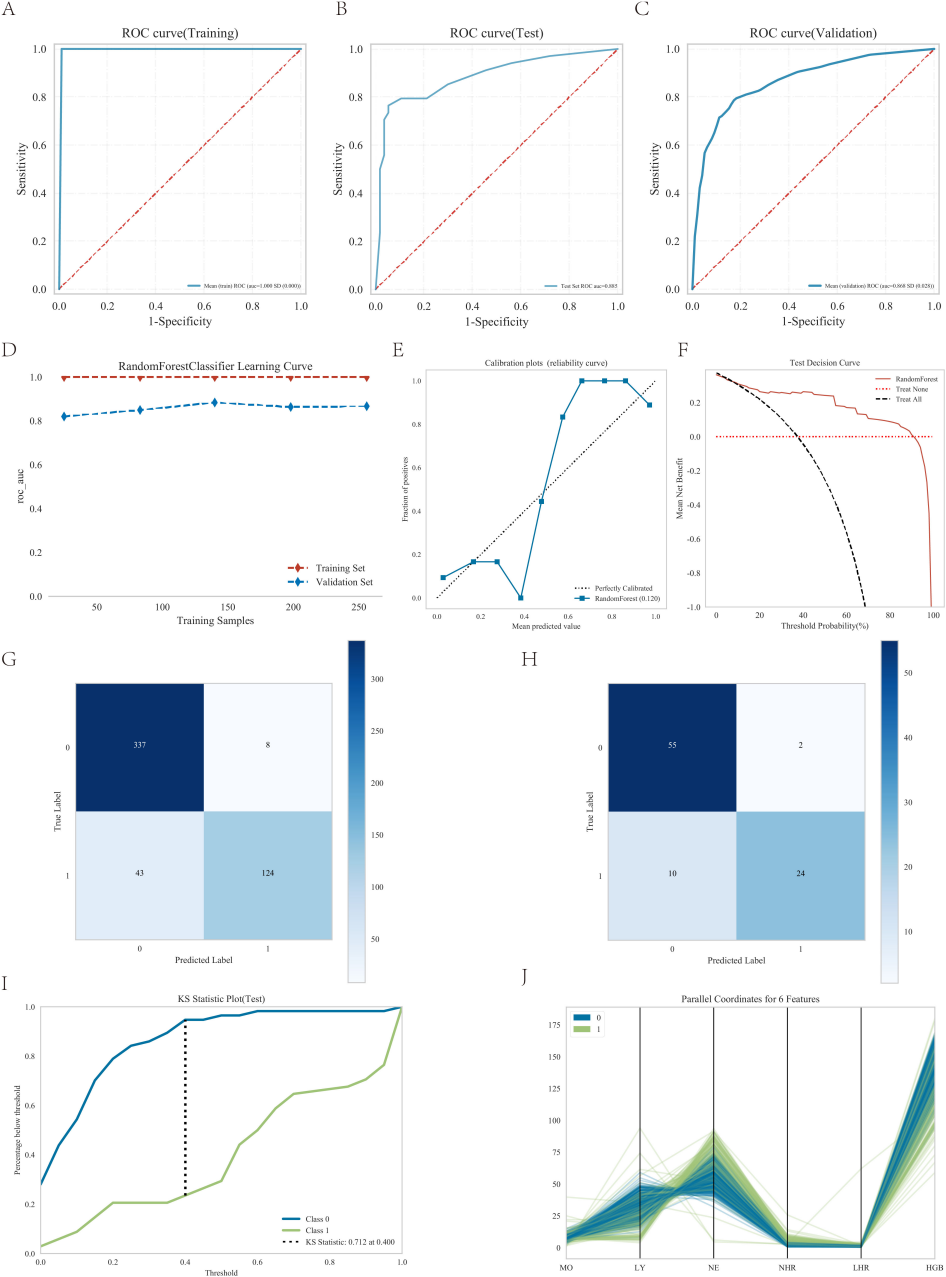
Performance of the predicted modes. **(A)** ROC curve for the test cohort; **(B)** ROC curve for the test cohort; **(C)** ROC curve for the validation cohort; **(D)** AUC for the test cohort; **(E)** calibration curve analysis; **(F)** decision curve analysis; **(G)** confounding matrix for the training set; **(H)** confounding matrix for the test set; **(I)** KS statistic plot for the test cohort; **(J)** parallel coordinates for 6 features.

As shown in Figures 5A–C and Table 3, the AUC values of the test cohort were comparable to those of the validation cohort. Figure 5D shows comparable performance between validation and training cohorts, demonstrating the model’s appropriate fit without overfitting. As shown in Table 3, the model achieved >70% accuracy, sensitivity, and specificity in the test cohort.

**TABLE 3:** Diagnosis effect of random forest (RF) models in the test and validation cohort.

Cohorts	AUC	Cutoff	Accuracy	Sensitivity	Specificity	Positive predictive value	Negative predictive value	F1
Training	1.000	0.500	1.000	1.000	1.000	1.000	1.000	1.000
Validation	0.868	0.500	0.838	0.665	0.922	0.810	0.853	0.724
Testing	0.885	0.500	0.868	0.735	0.947	0.893	0.857	0.806

Furthermore, the calibration curve showed strong agreement between actual and predicted probabilities, with points closely following the diagonal (Figure 5E), while the decision curve analysis confirmed the model’s substantial clinical utility (Figure 5F), thereby validating the excellent performance of the random forest model.

**FIGURE 6:**
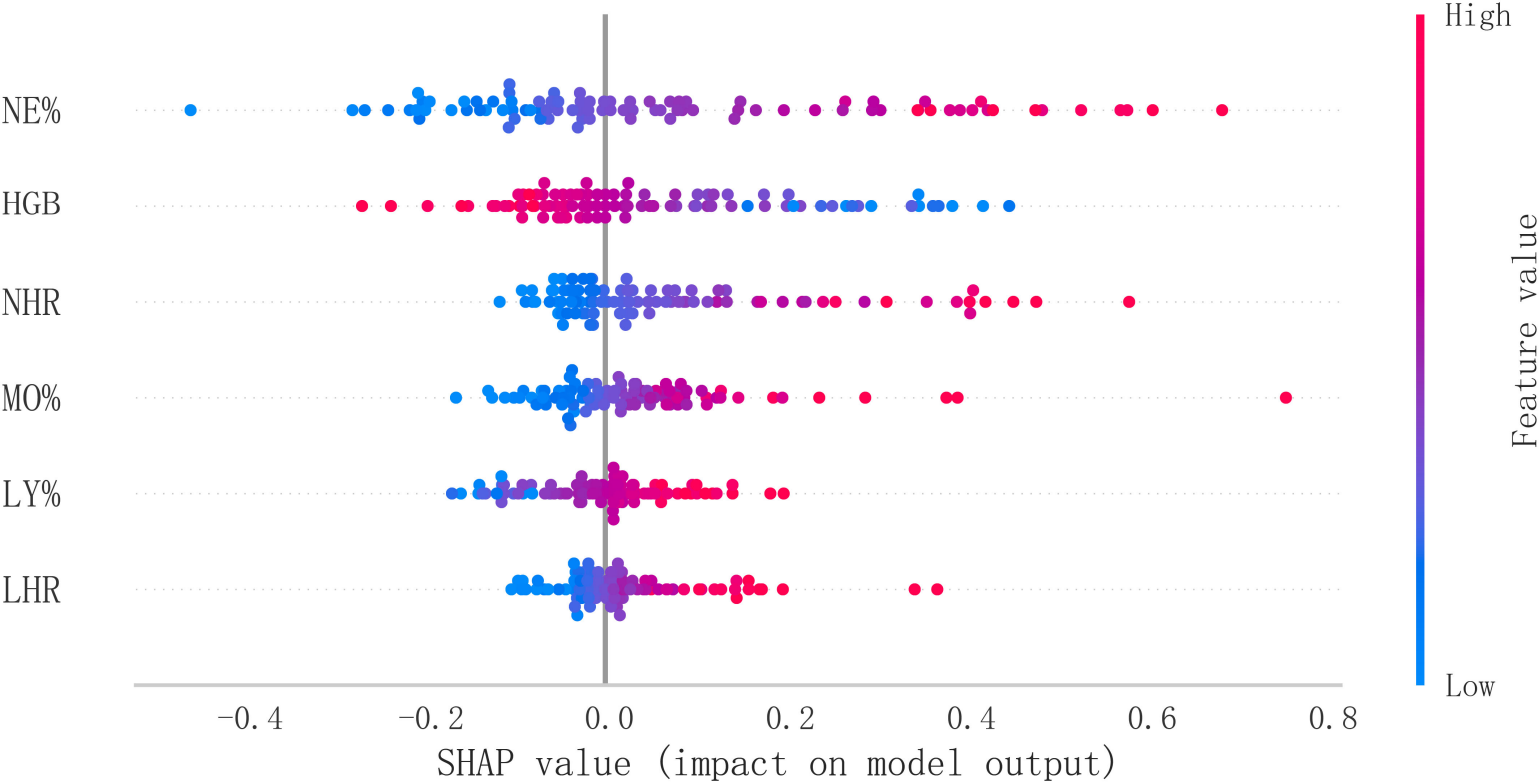
Overall SHAP explanations. SHAP explanations, red color represents higher values for covariates, while blue represents lower values for covariates. The x-axis represents the change in the log probability of ILD.

The confusion matrix results demonstrated variations in model performance across different datasets. In the training set (Figure 5G). The model showed consistent performance across datasets with training set sensitivity of 74.3% and specificity of 97.7% (Figure 5G), compared to 70.6% sensitivity and 96.5% specificity in the testing set (Figure 5H). Figure 5I shows the KS statistic plot for the test cohort, and Figure 5J presents parallel coordinates for six features. Additionally, Figure 6 illustrates the SHAP values for all covariates that predict the probability of interstitial lung disease.

### 3.6 External validation of the random forest model

**FIGURE 7:**
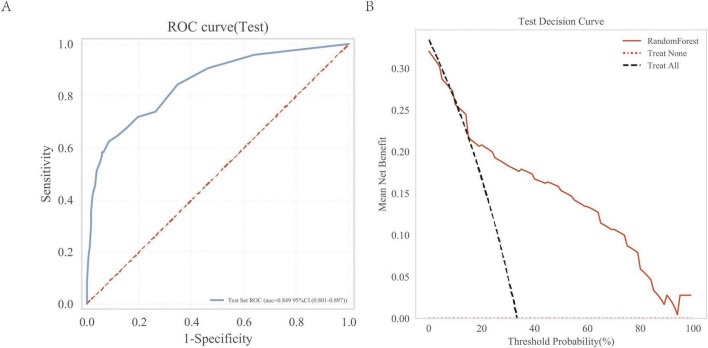
External independent test of the random forest (RF) model. **(A)** Subject operating characteristics (ROC) curves for the external independent testing; **(B)** Test decision curve for the external independent test cohort.

We evaluated the model using an independent external validation cohort of 288 patients from two additional medical centers. The model showed an AUC of 0.849 (Figure 7A) and demonstrated substantial clinical utility in decision curve analysis (Figure 7B).

### 3.7 Online prediction platform

**FIGURE 8:**
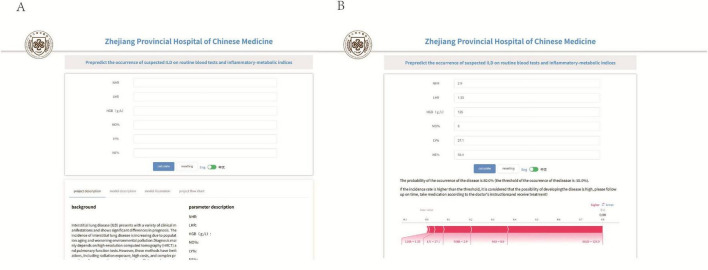
An online prediction tool **(A,B)** that predicts the probability of ILD based on the random forest (RF) model, according to the online page for six indicators to predict risk.

Based on the aforementioned analysis, we developed an online predictive platform to assist primary care clinicians in assessing interstitial lung disease risk in patients with suspected symptoms (Figure 8). This platform enables users to input six key blood biomarkers (NE%, LY%, MO%, HGB, LHR, NHR) to estimate disease probability. https://www.xsmartanalysis.com/model/list/predict/model/html?mid=26937&symbol=9175488Bp0Bm85Ap4DG1

## 4 Discussion

Interstitial lung disease (ILD) (9) represents a diverse group of disorders affecting the lung interstitium through inflammation and fibrosis. Its etiology is complex, involving environmental exposures, genetic factors, and autoimmune mechanisms. While clinical presentations vary, most ILD patients experience progressive dyspnea, dry cough, and declining pulmonary function. In advanced cases, these may lead to respiratory failure with potentially fatal outcomes. Although high-resolution computed tomography (HRCT) remains essential for ILD diagnosis (10), significant challenges exist, especially in early-stage disease detection. Diagnostic challenges are exacerbated in resource-limited settings by: (1) restricted HRCT availability, (2) insufficient physician training, and (3) lack of screening instruments. Consequently, developing reliable biomarkers and diagnostic association models would substantially enhance early ILD detection and risk assessment.

This study analyzed 891 patient records from Zhejiang Provincial Hospital of Traditional Chinese Medicine (including Hubin Branch and two others). It aimed to identify ILD-associated blood biomarkers and develop machine learning prediction models. Using LASSO regression and various machine learning algorithms, we selected key indicators including: neutrophil percentage (%), lymphocyte percentage (%), monocyte percentage (%), hemoglobin (g/dL), NHR and LHR. The random forest model showed superior performance (accuracy: 86.8%, AUC: 0.885) in both internal and external validation compared to other algorithms, establishing it as the optimal diagnostic association model. These findings equip clinicians with a machine-learning tool that leverages routine bloodwork to reliably flag the presence of ILD.

This study pioneers the integration of multiple blood-derived biomarkers with machine learning algorithms to enable early risk prediction and diagnosis of ILD. Our feature selection process, employing LASSO regression, revealed six clinically significant indicators: NE%, LY%, MO%, HGB, NHR, and LHR. While some of these parameters have been individually examined, their combined application represents a novel approach, particularly the innovative use of NHR and LHR ratios in ILD assessment.

The white blood cell count, particularly neutrophil levels, is closely associated with ILD development (9). Studies indicate that neutrophils promote fibrosis through the release of proteolytic enzymes MMP-9 and NETs (11). As part of the innate immune system, monocytes potentially contribute to ILD pathogenesis by differentiating into pulmonary macrophages (12, 13). Research has identified circulating hybrid TLR4M2 monocytes as potential biomarkers for progressive pulmonary fibrosis in SSc-ILD (14).

Hemoglobin, the crucial oxygen transport protein, may demonstrate reduced levels in ILD patients due to impaired alveolar ventilation and diffusion capacity. SLE-ILD patients show significantly lower hemoglobin levels than non-ILD counterparts (*P* < 0.05), with low hemoglobin levels being an independent risk factor (15). This hematologic pattern is also observed in other connective tissue disease-related ILDs (16, 17).

In addition to its role in lipid metabolism, HDL also has anti-inflammatory, antioxidant, and antifibrotic properties. Emerging evidence reveals its significant association with ILD onset, progression, and prognosis. Low HDL-C represents an independent risk factor for RA-ILD, while high levels appear protective (18). Decreased activity of the functional marker PON1 correlates with endothelial damage and increased DM-ILD risk (19).

The novel inflammatory-metabolic indices LHR and NHR, integrating monocyte/neutrophil (inflammatory markers) with HDL-C (anti-inflammatory/antioxidant marker) ratios, reflect the imbalance between inflammatory processes and repair mechanisms. These indices have demonstrated prognostic value in cardiovascular diseases, sepsis, periodontitis, and depression (20–23).

It is noteworthy that the AUC values for NE%, LY%, MO%, HGB, NHR and LHR were 0.752, 0.704, 0.603, 0.741, 0.793, and 0.567, respectively, while our model achieved an AUC of 0.864. This demonstrates that our model both improves the predictive performance of individual parameters and shows superior efficacy. Compared with the study by Qin et al. (8), our research not only validates the importance of routine hematological parameters in ILD but also identifies the significant role of novel derived parameters. Furthermore, our machine learning model significantly improves prediction accuracy. Notably, our study pioneers the development of an online prediction tool, providing valuable diagnostic support for primary care clinicians.

Our findings provide significant implications and important guidance for clinical practice. First, machine learning models enable more accurate identification of high-risk ILD patients, facilitating early intervention to improve outcomes. Second, the blood biomarkers identified in this study offer novel insights into ILD pathological mechanisms, particularly the discovery of LHR and NHR, which suggests the potential role of lipid metabolism and inflammatory responses in ILD. These findings both optimize the ILD diagnostic process and suggest new therapeutic targets for future treatment strategies. Furthermore, widespread adoption of our online prediction tool will enhance primary healthcare diagnostics for ILD, reducing errors and creating societal benefits.

This study has several limitations. First, our sample size remains relatively small despite using data from two centers. This may limit the results’ broad applicability. Second, the retrospective design introduces possible selection and information biases. Third, the models’ performance depends on data quality and feature selection, despite using multiple machine learning approaches. Fourth, the cohort of this study was dominated by CTD-ILD (87%), so the generalization ability of the model in other ILD phenotypes (such as IPF, HP, sarcoidosis, etc.) has not been verified. In subsequent studies, larger CTD-ILD specimens will be included to build a pure cohort training set, systematically evaluate the improvement of model performance, and further verify the incremental value of this dedicated tool in clinical decision-making. Finally, our online prediction tool requires additional validation before clinical implementation.

## 5 Conclusion

This study employed the lasso regression method to identify key biomarkers associated with interstitial lung disease, including neutrophil percentage (NE%), lymphocyte percentage (LY%), monocyte percentage (MO%), hemoglobin (HGB), LHR, and NHR. After evaluating eight different machine learning models, the random forest (RF) model demonstrated the best performance, showing exceptional predictive accuracy and clinical utility. The model performed excellently in both internal and external validations, indicating significant clinical application potential. Additionally, our developed web-based predictive tool offers primary care physicians an easy-to-use method for risk assessment.

## Data Availability

The datasets presented in this article are not readily available because the data can only be used for research purposes and not for commercial use. Requests to access the datasets should be directed to LC, 20163323@zcmu.edu.cn.
