# Supplementary material for: Identification of routine blood derived hematological and lipid indices in ILD through machine learning; a retrospective case-control study
Source: Front Med (Lausanne). 2025 Oct 9;12:1633713. doi: 10.3389/fmed.2025.1633713 (PMC12545033; doi:10.3389/fmed.2025.1633713)
Supplement: Supplementary file 2 [file Table_1.docx]

Supplementary Table 1 laboratory parameter definitions

| **Variable** | **Unit** | **Measurement method** |
| --- | --- | --- |
| **Hematology** |  |  |
| Leukocyte count (WBC) | 10⁹/L | Automated hematology analyzer (Mindray CAL 8000) |
| Neutrophils (NE) | 10⁹/L & % | Differential count from same analyzer |
| Lymphocytes (LY) | 10⁹/L & % | Differential count from same analyzer |
| Monocytes (MO) | 10⁹/L & % | Differential count from same analyzer |
| Eosinophils (EO) | 10⁹/L & % | Differential count from same analyzer |
| Basophils (BA) | 10⁹/L & % | Differential count from same analyzer |
| Hemoglobin (HGB) | g/L | Cyanmethemoglobin method |
| Red-cell count (RBC) | 10¹²/L | Impedance counting |
| Red-cell Distribution Width (RDW) | % | Calculation formula: RDW-CV (%) = (Standard deviation of red-cell volume ÷ Mean corpuscular volume, MCV) × 100 |
| Platelet count (PLT) | 10⁹/L | Impedance counting |
| Mean platelet volume (MPV) | fL | Optical platelet analysis |
| Platelet Distribution Width (PDW) | % | Calculation formula: PDW (%) = (Standard deviation of platelet volume ÷ Mean platelet volume, MPV) × 100 |
| **Lipid metabolism** |  |  |
| Triglycerides (TG) | mmol/L | Enzymatic colorimetric assay (GPO-PAP) after 12-h overnight fast |
| Total cholesterol (CHOL) | mmol/L | CHOD-PAP method |
| High-density lipoprotein cholesterol (HDL-C) | mmol/L | Direct homogeneous assay, polyethylene glycol-modified enzymes |
| Low-density lipoprotein cholesterol (LDL-C) | mmol/L | Calculated by Friedewald formula: LDL-C = CHOL − HDL-C − TG/2.2 (if TG < 4.5 mmol/L); otherwise measured directly |
